# Benford’s Distribution in Complex Networks

**DOI:** 10.1038/srep34917

**Published:** 2016-10-17

**Authors:** Mikołaj Morzy, Tomasz Kajdanowicz, Bolesław K. Szymański

**Affiliations:** 1 Institute of Computing Science, Poznań University of Technology, Poznań, 60–965, Poland; 2 Faculty of Computer Science & Management, Wrocław University of Science and Technology, 50–370 Wrocław, Poland; 3 Social and Cognitive Networks Academic Research Center, Rensselaer Polytechnic Institute, Troy NY 12180, USA.

## Abstract

Many collections of numbers do not have a uniform distribution of the leading digit, but conform to a very particular pattern known as Benford’s distribution. This distribution has been found in numerous areas such as accounting data, voting registers, census data, and even in natural phenomena. Recently it has been reported that Benford’s law applies to online social networks. Here we introduce a set of rigorous tests for adherence to Benford’s law and apply it to verification of this claim, extending the scope of the experiment to various complex networks and to artificial networks created by several popular generative models. Our findings are that neither for real nor for artificial networks there is sufficient evidence for common conformity of network structural properties with Benford’s distribution. We find very weak evidence suggesting that three measures, degree centrality, betweenness centrality and local clustering coefficient, could adhere to Benford’s law for scalefree networks but only for very narrow range of their parameters.

Benford’s law is a well-documented phenomenon describing the distribution of the most significant digit in many different datasets. Originally noticed by Newcomb^1^ and Benford^2^, it states that the probability of the most significant digit of a random element of a real-world numerical dataset being *d* is given by


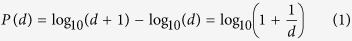


At first, Benford’s law seems very counter-intuitive. Why wouldn’t the leading digits be uniformly distributed in real-world datasets? Yet, this phenomenological law holds for an extraordinary diversity of datasets. Benford’s distribution has been observed in geophysical data^3^, such as distributions of lengths of rivers, areas of lakes, etc., in the distribution of auction prices on eBay^4^, or in the effects of introducing Euro currency in EU member states^5^. Recently, Benford’s law has been used in fraud detection^6^,^7^,^8^, to indicate vote counting manipulation during elections in the US^9^, Ukraine and Russia^10^ (although some researchers claim that Benford’s law is not the right tool to assess the veracity of elections^11^), and to disclose inconsistencies in census surveys^12^. The same distribution has been found in engineering where failure rates and mean-time-to-failure (MTTF) values of information systems closely follow the logarithmic pattern^13^. It has also been reported that several properties of complex networks (such as centrality indexes) obey Benford’s law as well^14^. Even more surprisingly, Benford’s law applies also if the numbers are multiplied by a constant, or expressed in a numeral system other than decimal. In other words, Benford’s law is both scale-invariant and base-invariant.

Benford’s law has intrigued both scientists and general population for over a century. There were many who claimed that it is an inherent property of the universe, an esoteric law of nature which applies to some datasets. It has not been helpful that the original discoverer of this logarithmic rule, American astronomer Simon Newcomb, following the infamous example of Pierre de Fermat, described his discovery as “evident”, without any explanation. His statement was simply that “The law of probability of the occurrence of numbers is such that all mantissae of their logarithms are equally likely”^1^. 60 year later, when Frank Benford, a physicist working at Corporate Research and Development Center of General Electric, assembled the collection of over 20 000 numbers from many different sources (atomic weights, population sizes, physical constants, street addresses, *Readers’ Digest* articles) and re-discovered the logarithmic distribution of the leading digit, he claimed that the phenomenon only applied to “anomalous” and “outlaw” numbers.

This does not mean that no serious attempts have been made to come up with a plausible explanation of the origins of Benford’s law. Raimi^15^ presents a thorough summary of previous works on the derivation of Benford’s law. He claims that the first robust statistical explanation of Benford’s law has been proposed by Pinkham^16^. The argument of Pinkham relied heavily on the scale-invariance property of Benford’s distribution. Today, it is widely accepted that another explanation, given by Hill^17^,^18^ and based on random sampling from a mixture of random distributions, is more correct. An analytical explanation based on the multiplicative nature of fluctuations has been proposed by Pietronero *et al*.^19^.

In this paper we examine whether structural properties of complex networks agree with Benford’s distribution. In order to present our findings, we introduce basic notions and definitions pertaining to network structural properties, and in particular, to centrality measures. Let *G* = 〈*V*, *E*〉 be a network with the set of vertices *V* = {*v*_1_, *v*_2_, …, *v*_*n*_} and the set of edges *E* = {(*v*_*i*_, *v*_*j*_):*v*_*i*_, *v*_*j*_ ∈ *V*}. Let *d*(*v*_*i*_) denote the *degree* of the vertex *v*_*i*_, i.e. the number of vertices adjacent to *v*_*i*_. Let *δ*(*v*_*i*_, *v*_*j*_) be the set of shortest paths between vertices *v*_*i*_ and *v*_*j*_ in the network *G*, and let *δ*_*k*_(*v*_*i*_, *v*_*j*_) be the set of shortest paths between vertices *v*_*i*_ and *v*_*j*_ which pass through the vertex *v*_*k*_. Finally, let Δ(*v*_*i*_, *v*_*j*_) denote the length of the shortest path between vertices *v*_*i*_ and *v*_*j*_. A *centrality measure* is a function 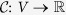 which assigns to each vertex a value representing the “importance” of the vertex in the network *G*. Of course, there are many different ways in which the importance of a vertex can be defined.

- *degree centrality C*_*D*_(*v*_*i*_) = *d*(*v*_*i*_) simply measures the number of vertices adjacent to the vertex *v*_*i*_. The assumption here is that a vertex is important if it is directly connected to many vertices in the network.
- *betweenness centrality* 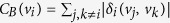 measures the number of shortest paths between any pair of vertices which pass through the vertex *v*_*i*_. This interpretation of importance highlights the influence of a vertex on communication pathways through the network.
- *closeness centrality* 
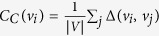
 measures the average distance from the vertex *v*_*i*_ to all other vertices in the network. According to this definition, a vertex is important if it can quickly communicate with all remaining vertices in the network.

Apart from these three centrality measures^20^, vertices in complex networks are commonly described using the *local clustering coefficient*. This feature describes the local neighborhood of a vertex, also known as the *egocentric network* of *v*_*i*_, which consists of the vertex *v*_*i*_, all its adjacent vertices, and all edges between these vertices. For a given vertex *v*_*i*_, its local clustering coefficient is defined as the number of edges existing in its egocentric network divided by the maximum number of edges which could exist in this egocentric network (i.e. the number of edges that would exist in a clique of equal size). Local clustering coefficient is a convenient measurement of the completeness of the local neighborhood of a vertex. Figure 1 illustrates centrality measures for vertices. This network has been introduced by Ulrik Brandes and it is the smallest network in which four different vertices attain the maximum value of degree, betweenness, closeness, and local clustering coefficient, respectively. For each of the discussed centrality measures the size and the intensity of color of each vertex correspond to the value of the centrality measure.

In this paper we search for Benford’s distribution in various characteristics of complex networks. We investigate both real world networks and artificial networks, generated from popular network models: Erdös-Rényi random network model, Watts-Strogatz small world network model, Albert-Barabási preferential attachment model, and the forest fire model. We compute the distributions of centrality measures and perform multiple tests of agreement of these distributions with Benford’s distribution. Quite surprisingly, we find that despite power law distributions of centrality measures, they do not conform to Benford’s distribution, with a notable exception of betweenness centrality, which, for many of the examined networks, exhibits signs of conformity with Benford’s distribution.

## Results

### Real world datasets

In our experiments we have used datasets from the Stanford Large Network Dataset Collection^21^, as well as the datasets used by Golbeck^14^ and by Zhong *et al*.^22^. Table 1 summarizes main characteristics of these datasets. Since there is no agreed-upon procedure of testing for the presence of Benford’s distribution in a dataset, for each of the considered networks we have performed 11 independent tests described in Section *Methods*. Each of these tests tries to establish the goodness of fit with Benford’s distribution based on a different criterion. We have observed that none of approximately 8000 distributions of structural properties of artificial and real word networks was able to pass more than 2 goodness of fit tests, with the notable exception of betweenness. Thus, for the purpose of the evaluation of results within the paper we have decided to use, as a local criterion of agreement with Benford’s distribution, the threshold of 2 passed goodness of fit tests.

Table 2 presents our findings, the last column contains the number of goodness of fit tests with positive results. Out of 15 real world networks only 5 networks have a structural property which passes the local criterion of agreement with Benford’s distribution, and this property is almost exclusively betweenness. Our local criterion is very lenient, should we have used a slightly more strict threshold, only two relatively small datasets (*facebook* and *twitter*) would have fulfilled the local criterion.

### Artificial datasets

Real world datasets are often incomplete, dirty, or biased by the harvesting method. The obvious lack of Benford’s distribution in structural properties of real world networks could be caused by the noise in real world data that distorted the outcomes of our analysis. To eliminate this possibility, we perform the analysis on artificial networks generated from a few popular generative network models. We have used the following artificial network models:

- *Erdös-Rényi random model*^23^ creates a network consisting of *n* vertices, and for each pair of vertices (*v*_*i*_, *v*_*j*_) an edge is created between them with the probability *p* (where *n* and *p* are the parameters of the model).
- *Watts-Strogatz small world model*^24^ creates a network of *n* vertices organized in a ring topology, where each vertex is connected to its *k* closest neighbors. After creating the initial ring each edge is randomly rewired with a very small probability *p*. Vertices in the resulting network tend to have similar degrees and their local clustering coefficients are an order of magnitude greater than in a random network. The rewiring process drastically changes the betweenness of a small number of nodes, which serve as bridges to remote parts of the network.
- *Albert-Barabási preferential attachment model*^25^ creates a network from an initial complete graph 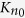 consisting of *n*_0_ vertices. Subsequent vertices are added sequentially, and each new vertex creates *k* edges. The probability of choosing a vertex *v*_*i*_ as the target vertex for a new edge is proportional to its current degree *d*(*v*_*i*_). The resulting network has a power law distribution of vertex degrees and vertex betweennesses.
- *forest fire model*^26^ also adds vertices sequentially. Upon arrival each vertex creates edges to *k* uniformly selected vertices, called *ambassadors*, and then adds more edges to neighbors of ambassadors with the *forward burning probability p*. The process continues recursively for each vertex to which an edge has been added.

We have generated 50 instances of networks for each artificial network model and each value of the model parameter, and for each model we have tested 10 different values of the main model parameter. Each network had a constant size of *n* = 1000 vertices and for each network we have computed four distributions: degree, betweenness, closeness, and local clustering coefficient. Altogether we have tested 4*10*50*4 = 8000 possible distributions for the agreement with Benford’s distribution. Model parameters have been uniformly selected from the following ranges:

- *Erdös-Rényi random model*: random edge probability *ep* ∈ [0.001, 0.01]
- *Watts-Strogatz small world model*: random edge rewiring probability *rp* ∈ [0.01, 0.05]
- *Albert-Barabási preferential attachment model*: power law exponent *ac* ∈ [1, 3]
- *forest fire model*: forward burning probability *fb* ∈ [0.01, 0.25]

Table 3 presents the results of our experiments on artificially generated networks. Most of tests failed to discover Benford’s distribution in any of complex networks’ structural properties, and only 5 tests produced any positive results. The number of positive results for each test is presented in Table 4. Despite very weak evidence for the presence of Benford’s distribution in artificial networks, both Mantissa Arc test and the *χ*^2^ test signal the conformity with Benford’s distribution in networks generated using the preferential attachment process. These networks are known to have a power law distribution of betweenness^27^ and local clustering coefficient^28^. As has been shown before^29^, a distribution is more likely to adhere to Benford’s distribution if it resembles a survival distribution, i.e. it puts most of its mass on small values of the random variable, and power law distribution fulfills this condition. The Albert-Barabási preferential attachment model generates networks with power law distributions of vertex degrees as well. Yet, on the first glance surprisingly, this structural feature is never found to conform to Benford’s distribution. However, the analysis of network properties with the nodes distributed according to the power law provides an explanation.

For a series of elements with power law distribution, the probability of series element having the given value is a decreasing function of such value with the maximum probability at the minimum value in the series. An immediate conclusion is that only the series with the minimum value having the leading digit of 1 has a chance to conform to Benford’s law. There is also a restriction on the power law exponent, which cannot be too large. As we have empirically checked, it must be no larger than 1.25 when the series has the minimum and maximum values of 1 and 10 respectively. This range is even smaller for larger ranges of the minimum and maximum values. In summary, only series with the minimum value in the range [10^*k*^, 10^*k*+1^), *k* = 1, …, the maximum value around 10^*k*+*m*^, *m* = 1, … and with the power law exponent in the range [1, 1.25] may have distribution of its element values resembling Benford’s distribution.

This analysis directly applies to the degree centrality measure for networks with power law distribution of node degrees. Thus, only a network with minimum degree in the range [10^*k*^, 10^*k*+1^), *k* = 0, 1, …, the natural cut off around 10^*k*+*m*^, *m* = 1, … and with the power law exponent in the range [1, 1.25] may have degree centrality measure distributed according to Benford’s law. Betweenness centrality for networks with power law distributed node degrees is also power law distributed^27^. The minimum number of shortest paths between any two nodes passing through the given node is *n* − 1. Hence, only a network with the number of nodes in the range [10^*k*^ + 1, 10^*k*+1^], *k* = 0, 1, …, the natural cut off around 10^*k*+*m*^, *m* = 1, … and with the power law exponent of betweenness centrality between 1 and 1.25 may have betweenness centrality measure distributed according to Benford’s law. Also local clustering coefficient of networks with power law distribution of node degree has the power law distribution^28^. Here, only when the minimum non-zero local clustering coefficient has its first significant digit being 1 and the power law exponent of the distribution of local clustering coefficients is in the range [1, 1.25] this measure may obey Benford’s law.

Finally, a similar analysis of node degree distribution for the Erdös-Rényi random network model may start with an observation that the node degree with the highest probability of appearing in the network is the integer closest to *p*(*n* − 1) (where *p* is the probability of having an edge between any pair of nodes) and it must have the leading digit of 1. On the other hand, the width of the distribution is narrow, about the square root of the average degree, so it is too narrow to reach on the right of the average degree to the degrees with digits larger than 2. Thus, the frequencies for such larger digits have to come from the range left of the average degree. Hence the frequencies will be increasing for digits growing from 3 to 9, while in Benford’s distribution those frequencies are decreasing. The conclusion is that an Erdös-Rényi network may have the node degree distribution resembling Benford’s distribution only if its average degree is close to 1, in agreement with our results.

## Discussion

Our analysis shows that previously reported presence of Benford’s distribution in complex networks^14^ is not supported by the rigorous set of tests that we conducted. A thorough examination using several different statistical tools does not indicate the presence of Benford’s distribution in complex networks. These results allow us to conclude that Benford’s distribution is not commonly present in the structural properties of either empirical or artificial complex networks. We also present here theoretical analysis of networks with power law distribution of node degrees and measures of degree centrality, betweenness centrality, and local clustering coefficient. The analysis demonstrates that for only narrow ranges of the parameters of the power law distribution, specifically the minimum degree, the natural cut off and the power law exponent, the distributions of the considered measures may resemble Benford’s distribution.

The main practical conclusion that can be drawn from our results is that Benford’s Law cannot be used to check the correctness of structural properties of complex networks. However, for the networks with power law distributed node degrees, we show that the distribution of leading digits of these three measures is well defined by the parameters of power law distribution. So these easy to establish distributions can be used instead of Benford’s distribution to discover fraud, incompleteness or manipulation of network structure and such applications will be the subject of our future work.

## Methods

The literature provides several methods of testing the conformity of a given distribution with Benford’s distribution. These methods are highly dependent on the area of application; different protocols are used when analyzing financial results, voting registers, or network intrusion records. For instance, Nigrini and Miller^30^ advocate the use of second order tests for financial data diagnostics (testing frequencies of leading digits of differences between ranked values instead of values themselves) claiming that this method is superior when rounding of data occurs. Other tests include the Distortion Factor Model^31^ and the Bayesian approach proposed by Ley^32^. In order to perform a thorough verification of the presence of Benford’s distribution in complex networks structural features we employ 11 different tests, summarized below. We have used two R packages, BenfordTests^33^ and benford.analysis^34^. For each of the performed tests we reject the null hypothesis for p-value ≤ 0.05.

- *χ*^2^ *test*: Pearson’s chi-square goodness of fit test with the statistic defined as 
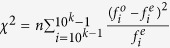
, where 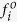 is the observed frequency of the digit *i*, and 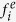 is the expected frequency of the digit *i*. The null hypothesis is that there is no difference between observed and expected frequencies.
- *Mean Absolute Deviation (MAD)*: the average deviation of the actual digit distribution from the expected Benford’s distribution, this statistic is defined as 
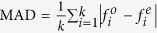
. We follow the suggestion of Nigrini^35^ and define MAD ≤ 0.0012 as close conformity, MAD ∈ [0.0012, 0.0018] as acceptable conformity, MAD ∈ [0.0018, 0.0022] as marginally acceptable conformity, and MAD ≥ 0.0022 as non-conformity to Benford’s distribution.
- *Mantissa Arc Test (MAT)*: the test computes the center of mass of a set of mantissae distributed on a unit circle. For a number *x* its coordinates on a circle are defined as follows: 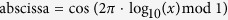, 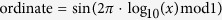. If the mantissae of a set of numbers {*x*_1_, *x*_2_, …, *x*_*n*_} are uniformly distributed on the circle, the center of the mass, also known as the *mean vector*, is at (0, 0), in other cases it will be at the distance of *L*^2^ from the center of the circle. The MAT test defines the following test statistics: 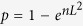 and this statistic is checked for significance against the *χ*^2^ distribution with 2 degrees of freedom. The *MAT* test has been first proposed by Alexander^36^.
- *Distortion Factor*: proposed by Nigrini^35^, this test compares the actual mean of the set of numbers with the mean expected for a Benford’s set of the same size using the standard Z-statistic.
- *Pearson’s r*: traditional Pearson’s product-moment correlation coefficient measuring the linear correlation between the observed frequency of digits and the frequency of digits expected in Benford’s distribution.
- *Kolmogorov-Smirnov test*: traditional test of the distance between cumulative distributions, with the test statistic defined as 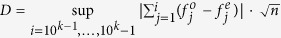. The result of the test is determined by the p-value of the D statistic.
- *Freedman-Watson Test*: a test to compare discrete distributions, its statistic is defined as

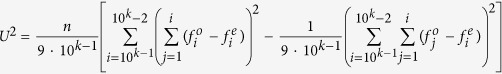


The result of the test is determined by the p-value of the *U*^2^ statistic.

- *Chebyshev Distance Test*: a simple maximum norm statistic defined as 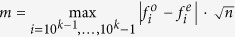. The result of the test is determined by the p-value of the *m* statistic.
- *Euclidean Distance Test*: performs a goodness of fit test based on the Euclidean distance between the observed and the expected digit distributions, test statistic is 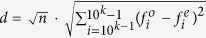. The result of the test is determined by the p-value of the *d* statistic.
- *Judge-Schechter Mean Deviation Test*: a goodness of fit test based on the deviation of mean digits, with the test statistic defined as 
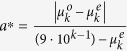
, where 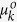 is the observed mean of the chosen *k* number of digits, and 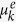 is the expected mean should the sample conform to Benford’s distribution. The test statistic a* under the null hypothesis has a truncated normal distribution, a* ~ *N*_*T*_(*μ* = 0, *σ* = *σ*_*B*_, *a* = 0, *b* = ∞).
- *Joenssens* 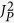 *Test*: a sign-preserving squared correlation test between the observed distribution and Benford’s distribution, with the test statistic defined as 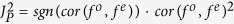. The result of the test is determined by the p-value of the 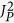 statistic.
- *Hotelling T*^2^ *Test*: a generalization of the Student’s t statistic to a multivariate case, this test uses the following statistic: 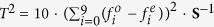, where **S** is the pooled covariance matrix. Under the null hypothesis the *T*^2^ statistic follows the F-distribution and the result of the test is determined by the p-value of the *T*^2^ statistic.

**Figure 2:**
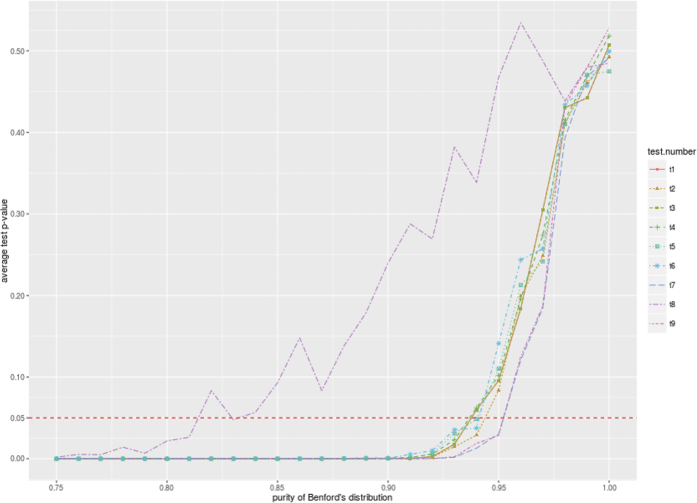
Average p-values of tests for different levels of Benford’s distribution purity.

Having used so many statistical tests to verify the goodness of fit with Benford’s distribution, we need to establish the sensitivity of tests and their mutual correlation. A simple way to do this is to run a suite of tests on data with varying degree of conformity with Benford’s distribution and to compute the p-values of these tests. In this experiment we use the following protocol. For each data point we create 50 random samples of 10 000 numbers, and we run all of the above tests on each sample. Then, we compute the average p-value for each test over these 50 samples. There are 100 data points, each representing a different mixture of Benford’s and normal distributions. Summary of these two distributions are presented in Table 5. Initially, all 10 000 numbers were drawn from the normal distribution, and in each step 1% of the sample is replaced by the numbers drawn from Benford’s distribution. Figure 2 shows the average p-values (ordinate) depending on the pureness of Benford’s distribution (abscissa). Most of the tests behave very similarly and reject the null hypothesis of the presence of Benford’s distribution until the distribution is 95% pure, while Freedman-Watson U-squared test and the Mantissa Arc test are slightly more conservative. The only exception is the Judge-Schechter Mean Deviation test, which signals the presence of Benford’s distribution already at the 82% pureness threshold.

In each test the null hypothesis states that the given set of numbers follows Benford’s distribution. Assuming the standard rejection threshold of the null hypothesis at p-value ≤ 0.05 level, Table 6 presents the average purity of Benford’s distribution accepted by each test. As can be seen, all tests (except for the Distortion Factor and the Judge-Schechter Mean Deviation tests) behave in a very coherent way, requiring a strong goodness of fit before accepting the null hypothesis. These results allow us to conclude that Benford’s distribution is not present in the structural properties of either empirical, or artificial complex networks. We also present here the analysis of networks with power law distribution of node degrees and measures of degree centrality, betweenness centrality, and local clustering coefficient. The analysis demonstrates that for only narrow ranges of the parameters of the power law distribution, specifically the minimum degree, the natural cut off, and the power law exponent, the distributions of the considered measures may resemble Benford’s distribution.

## Additional Information

**How to cite this article**: Morzy, M. *et al*. Benford’s Distribution in Complex Networks. *Sci. Rep.* **6**, 34917; doi: 10.1038/srep34917 (2016).

## Figures and Tables

**Figure 1:**
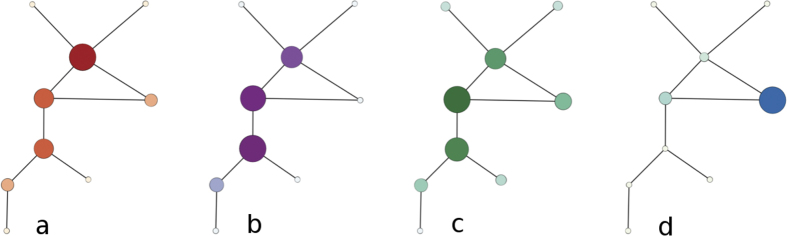
Centrality measures (**a**) degree (**b**) betweenness (**c**) closeness (**d**) clustering coefficient.

**Table 1:** Real world datasets (the sets used by Golbeck^14^ are marked with an ^†^ after their name).

Name	Description	Vertices	Edges
*amazon*	product co-purchase network	262 111	1 234 877
*citations*	paper citation network	27 770	352 807
*dblp*	scientific collaboration network	317 080	1 049 866
*enron*	email communication network	36 692	367 662
*facebook*^†^	friend counts	18 298	88 234
*google*+^†^	social circles network	107 614	30 494 866
*gnutella*	peer-to-peer network	36 682	88 323
*livejournal*^†^	friendship network	2 793 657	6 898 682
*pinterest*^†^	followers counts	67 648 287	67 648 287
*physics*	scientific collaboration network	12 008	237 010
*slashdot*	friendship network	82 168	948 464
*stanford*	website hyperlink network	281 903	2 312 497
*twitter*^†^	social circles network	81 306	2 420 766
*wikipedia*	adminship voting network	7115	103 689
*youtube*	friendship network	1 134 890	2 987 624

**Table 2:** Real world network properties which pass at least 2 goodness of fit tests.

Dataset	Measure	No. of passed tests
*citations*	degree	2
*citations*	betweenness	3
*enron*	betweenness	2
*facebook*	betweenness	7
*physics*	betweenness	2
*twitter*	betweenness	11

**Table 3:** Artificial network properties which pass at least 2 goodness of fit tests.

Model	Parameter	Measure	No. of passed tests
preferential.attachment	1.00	clustering	2
preferential.attachment	1.22	clustering	2
preferential.attachment	1.44	clustering	2
preferential.attachment	1.67	clustering	2
preferential.attachment	1.89	clustering	2
preferential.attachment	2.11	clustering	2
preferential.attachment	2.33	betweenness	2
preferential.attachment	2.33	clustering	2
preferential.attachment	2.56	betweenness	2
preferential.attachment	2.56	clustering	2
preferential.attachment	2.78	betweenness	2
preferential.attachment	2.78	clustering	2
preferential.attachment	3.00	betweenness	2
preferential.attachment	3.00	clustering	2
random.graph	0.001	clustering	2
random.graph	0.001	clustering	2
random.graph	0.001	clustering	2
small.world	0.001	betweenness	2

**Table 4:** Number of accepted goodness of fit tests from 60 real-world and 320 artificial network centrality measures distributions.

Mantissa Arc test	21
*χ*^2^ test	17
Judge-Schechter Mean Deviation test	11
Joenssen’s 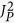 test	8
Distortion Factor	1

**Table 5:** Summary of distributions used in tests comparing goodness of fit.

Distribution	Min.	1st Qu.	Median	Mean	3rd Qu.	Max.
Normal	0.001028	2.676	3.981	4.031	5.335	11.69
Benford	1	1.804	3.202	3.927	5.645	9.996

**Table 6:** Average purity of Benford’s distribution accepted by each test.

Test	Test number	Average Purity
Chi-Square Test for Benford Distribution	t1	0.97
Euclidean Distance Test for Benford Distribution	t2	0.97
Joint Digits Test	t3	0.97
JP-Square Correlation Statistic Test for Benford Distribution	t4	0.97
K-S Test for Benford Distribution	t5	0.97
Chebyshev Distance Test for Benford Distribution	t6	0.97
Freedman-Watson U-squared Test for Benford Distribution	t7	0.98
Judge-Schechter Mean Deviation Test for Benford Distribution	t8	0.91
Mantissa Arc Test	t9	0.98
Distortion Factor	t11	0.79
